# Severe bilateral panuveitis during melanoma treatment by Dabrafenib and Trametinib

**DOI:** 10.1186/s12348-015-0049-9

**Published:** 2015-06-09

**Authors:** Dafina Draganova, Joseph Kerger, Laure Caspers, François Willermain

**Affiliations:** Department of Ophthalmology, Saint Pierre University Hospital, rue Haute 322, Brussels, 1000 Belgium; Jules Bordet Institute, Boulevard de Waterloo 121, Brussels, 1000 Belgium

**Keywords:** Dabrafenib, Trametinib, Uveitis, Serous retinal detachment, Chorio-retinal folds

## Abstract

**Background:**

We report a case of severe bilateral panuveitis during melanoma therapy with a combination of Dabrafenib, a B-raf (BRAF) inhibitor, and Trametinib, a mitogen/extracellular signal-regulated kinase (MEK) inhibitor. Both of these drugs are effectors in the mitogen-activated protein kinase (MAPK) pathway, which plays an important role in the physiopathology of melanoma. Dabrafenib and Trametinib have shown improved survival of patients with metastatic melanoma but they have also been associated with the development of uveitis.

**Findings:**

Our patient was a 55-year-old woman with metastatic melanoma who presented with sudden onset of bilateral painless visual loss. She had been treated with Dabrafenib and Trametinib. Trametinib was discontinued at the onset of symptoms but there was no improvement. Ophthalmological examination revealed severe bilateral non-granulomatous panuveitis, with choroidal thickening, chorio-retinal folds, and multiple serous retinal detachments (SRDs). Topical corticosteroid treatment was initiated, and Dabrafenib was discontinued. A good response was obtained with a recovery of visual acuity of 20/25 on both eyes and an almost complete resolution of the SRDs.

**Conclusions:**

This case highly suggests that MAPK pathway inhibition can lead to severe uveitis. Dabrafenib and Trametinib could have both played a role in inducing the disease. Further studies are needed to evaluate the possible role of the combination of these drugs in inducing uveitis and SRD.

## Findings

### Introduction

Medications are recognized as an increasingly common cause for uveitis [1]. In this article, we report a case of severe bilateral panuveitis during melanoma therapy with a combination of Dabrafenib, a B-raf (BRAF) inhibitor, and Trametinib, a mitogen/extracellular signal-regulated kinase (MEK) inhibitor. Progress in pharmacological research has led to the development of novel, more effective, and more specific drugs for melanoma. Some of them could be responsible for side effects, such as uveitis. The mitogen-activated protein kinase (MAPK) pathway plays a key role in the development of melanoma. Mutations in effectors of this pathway, such as BRAF and MEK, lead to increased survival and proliferation of melanoma cells [2]. Targeting these molecules has led to the development of promising drugs. Vemurafenib and Dabrafenib are BRAF^V600^ inhibitors licensed for the treatment of adults presenting the BRAF^V600E^ mutation and nonresectable or metastatic melanoma [2]. Trametinib is a MEK inhibitor approved for the same indication, as well as for a combination with Dabrafenib [2]. A combination of these molecules has shown an improved response rate in patients with metastatic melanoma [2]. A recent publication by Guedj et al. [3] described a series of seven patients who developed Vemurafenib-induced bilateral non-granulomatous anterior uveitis. Vemurafenib-induced uveitis was also reported by Choe et al. [4]. Another article describes a severe bilateral vitritis and optic disk leakage occurring 3 weeks after starting combined treatment with Dabrafenib and Trametinib, with resolution 6 weeks after the cessation of both drugs [5]. The large multicentre trials for the approval of Dabrafenib report the occurrence of 1 % of uveitis of unspecified severity [6]. The occurrence of serous retinal detachments (SRDs), once associated with moderate anterior uveitis, has been reported in Trametinib therapy [7,8]. Uveitis was resolved on topical therapy without the cessation of Trametinib.

### Case report

**Fig. 1:**
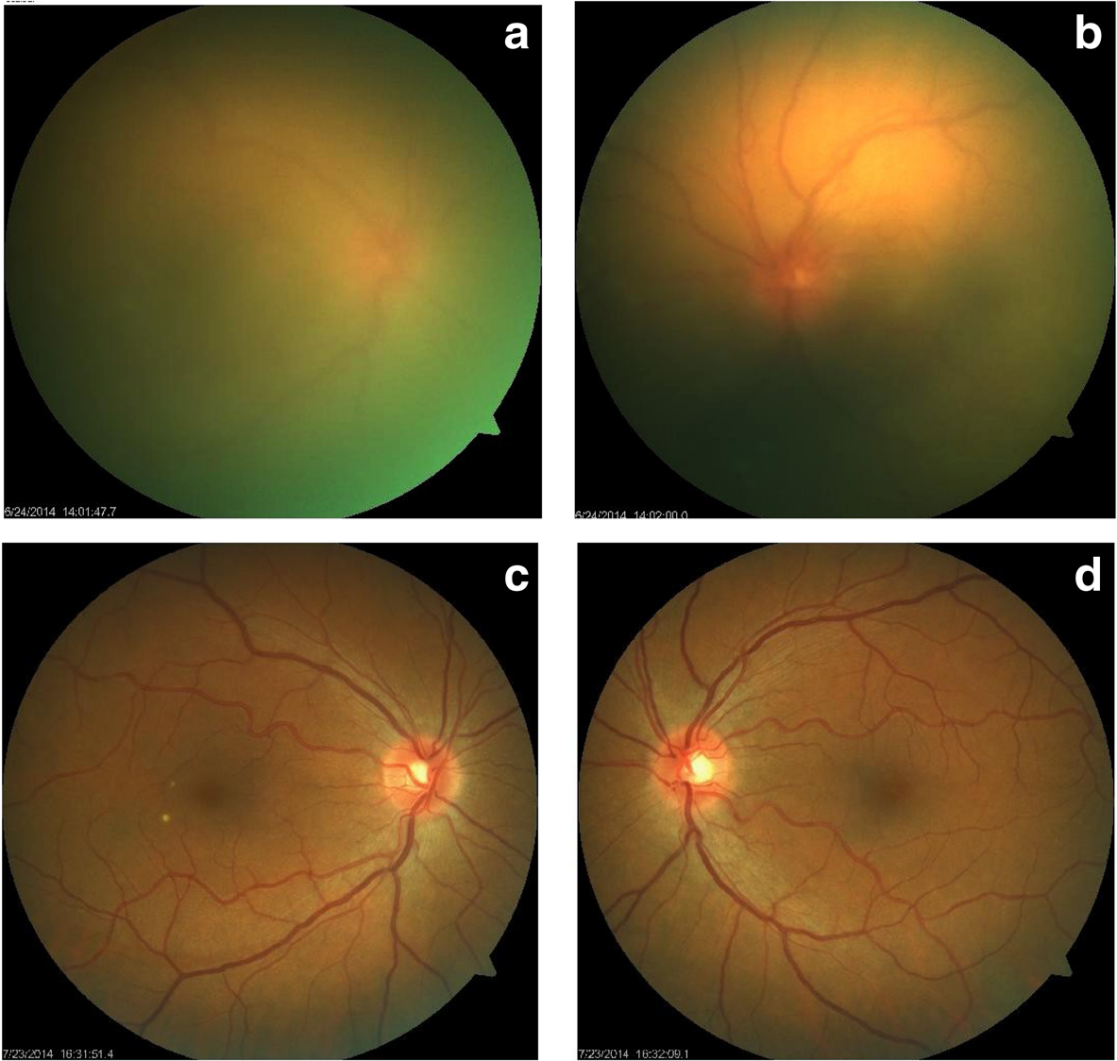
Color fundus photographs. **a**, **b** *Upper panel*: at presentation, fundus details are masked by anterior fibrin and vitreous haze resulting from severe inflammation, but optic disk hyperhemia can be detected on the left side. The superonasal round yellow area on the left side is an artifact. **c**, **d** *Lower panel*: 1 month after drug arrest, resolution of the inflammation allows normal fundus visualization including normal optic disks

**Fig. 2:**
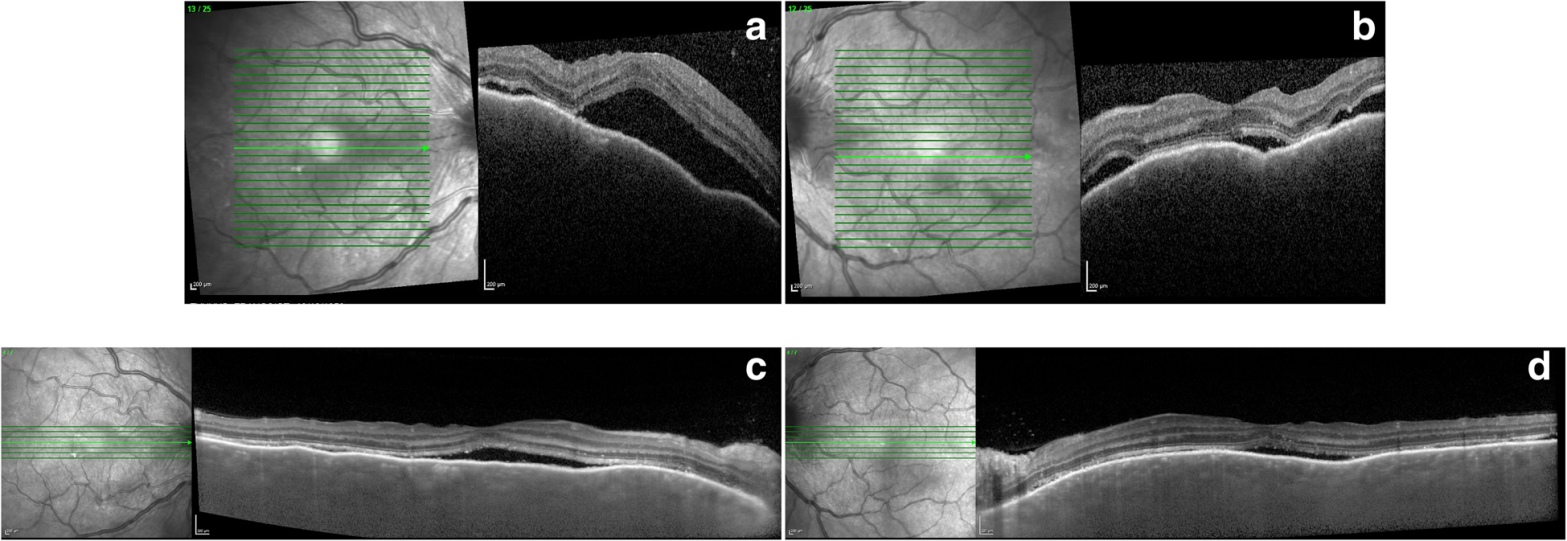
HR-OCT. **a**, **b** *Upper panel*: at presentation: multiple serous retinal detachments, loss of normal choroidal vascular architecture, and chorio-retinal folds are observed. **c**, **d** *Lower panel*: 1 month after drug arrest, there is an almost complete resolution of the serous retinal detachments and of the chorio-retinal folds

Our patient was a 55-year-old woman with metastatic cutaneous melanoma of unknown primary site who presented in June 2014 with sudden onset of bilateral painless visual loss. She had been treated with Dabrafenib 150 mg twice daily since November 2013 and Trametinib 2 mg once daily was added in April 2014. Trametinib was discontinued at the onset of symptoms by her oncologist, but there was no improvement after 10 days. The patient’s visual acuity was hand movements OD and counting fingers OS. Slit lamp examination revealed the following symmetric findings: no keratic precipitates, anterior chamber cells 2+ with fibrin, synechiae on 360°, and vitritis. Fundus details on both eyes were masked by anterior fibrin and vitreous haze resulting from severe inflammation, but optic disk hyperhemia could be detected on the left side and some chorio-retinal folds were visible on both eyes (Fig. 1, upper panel). Fluoangiography showed bilateral hot disks. However, due to the intensity of the inflammation, it was of insufficient quality to reveal other details. HR-OCT showed thickening and loss of normal choroidal vascular architecture, presence of chorio-retinal folds and multiple SRDs on both eyes (Fig. 2, upper panel). Uveitis work-up including blood work-up, chest X-ray, and a tuberculin skin test was negative. Topical corticosteroid treatment (one drop per hour) was initiated, and Dabrafenib was discontinued in agreement with the oncologist. A good response was obtained with a recovery of a visual acuity of 20/25 on both eyes, no detectable inflammation, and an almost complete resolution of the SRDs 1 month later (Figs. 1 and 2, lower panel). Further follow-up was impossible because the patient sadly passed away in September 2014.

### Discussion

To the best of our knowledge, this case report is the first to describe the simultaneous occurrence of severe non-granulomatous uveitis, SRDs, and chorio-retinal folds during combined Dabrafenib and Trametinib treatment. Even though the most common causes of uveitis are inflammatory or infectious disorders, medication side effects should always be considered in the differential diagnosis. There are several drugs for which the clinical experience is sufficient to establish a “definite,” “probable,” or “possible” association with uveitis using an algorithm proposed by Naranjo and associates [9]. Other more recent drugs, such as MAPK pathway inhibitors, have been described as inductors of uveitis in several independent publications. Currently, the best-documented cases are those presented in the series of Guedj et al. and Choe et al., concerning Vemurafenib [3]. Our case strongly suggests that BRAF inhibitor-induced uveitis is not restricted to Vemurafenib but can also occur with Dabrafenib. Our patient also received Trametinib, a MEK inhibitor that could have played a role in inducing the disease. Even though interruption of this medication did not result in clinical improvement, we cannot be certain that Dabrafenib alone could have caused the uveitis. In conclusion, it is probable that MAPK pathway inhibition can lead to severe uveitis. Further studies are needed to identify the patients who are at risk of developing this side effect, as well as to explore the possible role of the combination of several MAPK inhibitors in the induction of uveitis and SRD.

### Consent

Approval for the publication of this report and any accompanying images was obtained from the ethics committee of Jules Bordet Institute since the patient passed away before the request of informed consent.

## Abbreviations

BRAF: B-raf
MAPK: mitogen-activated protein kinases
MEK: mitogen/extracellular signal-regulated kinase
SRD: serous retinal detachment
